# Supplementary material for: Environmental DNA illuminates the darkness of mesophotic assemblages of fishes from West Indian Ocean
Source: PLoS One. 2025 May 22;20(5):e0322870. doi: 10.1371/journal.pone.0322870 (PMC12097626; doi:10.1371/journal.pone.0322870)
Supplement: S1 Fig — (DOCX) [file pone.0322870.s001.docx]

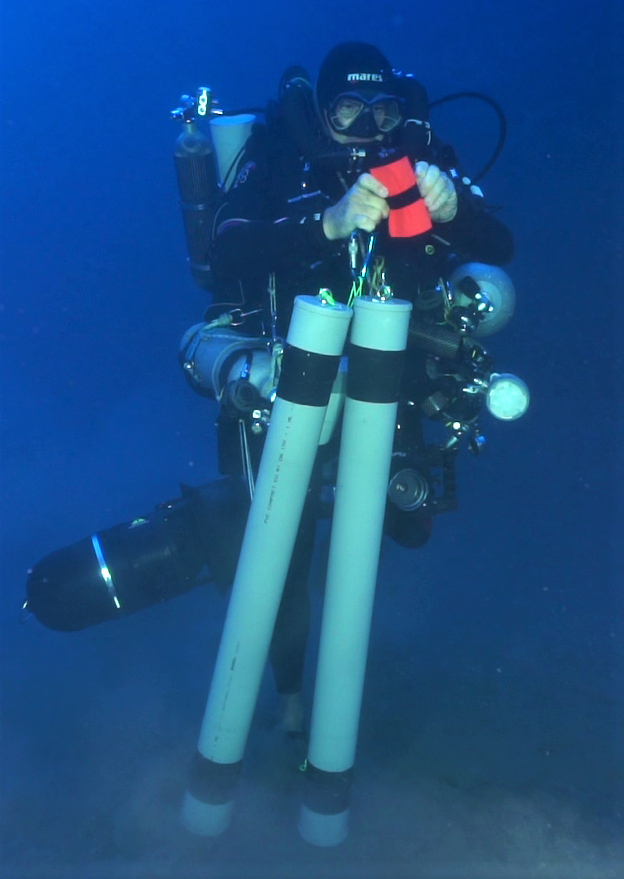


**S1 Fig.** **Mesophotic water sample collection with 8-liter sampling bottles developed for this study.**
