# Supplementary material for: Environmental DNA illuminates the darkness of mesophotic assemblages of fishes from West Indian Ocean
Source: PLoS One. 2025 May 22;20(5):e0322870. doi: 10.1371/journal.pone.0322870 (PMC12097626; doi:10.1371/journal.pone.0322870)
Supplement: S2 Table — (DOCX) [file pone.0322870.s002.docx]

**S2 Table.** **Data to characterize the environment at each sampled station.**

| ID | Island | Latitude | Longitude | Depth (m) | Slope | Rugosity level | Dominant substrate | Coastal distance (m) | Water volume filtered (L) |
| --- | --- | --- | --- | --- | --- | --- | --- | --- | --- |
| MesoRun307 | La Réunion | 21,15618 | 55,84092 | 93 | Low | 3 | Coral | 820 | 8 |
| MesoRun305 | La Réunion | 21,17538 | 55,27256 | 97 | Low | 2 | Meso-community | 1450 | 8 |
| MesoRun306 | La Réunion | 21,20383 | 55,27436 | 80 | Low | 1 | Coral | 660 | 8 |
| MesoRun312 | La Réunion | 21,17538 | 55,27256 | 96 | Low | 2 | Meso-community | 1450 | 8 |
| MesoRun308 | La Réunion | 21,11783 | 55,78518 | 93 | Low | 1 | Mud | 660 | 8 |
| MesoRun311 | La Réunion | 21,1207 | 55,78714 | 81 | High | 3 | Mud | 500 | 8 |
| MesoRun309 | La Réunion | 21,17577 | 55,27213 | 107 | Low | 2 | Meso-community | 1500 | 8 |
| MesoRun310 | La Réunion | 21,12242 | 55,80194 | 78 | Low | 1 | Mud | 585 | 8 |
| MesoMay01 | Mayotte | 12,812283 | 45,29595 | 83 | High | 2 | Meso-community | 1000 | 8 |
| MesoMay02 | Mayotte | 12,868705 | 45,270244 | 68 | Low | 1 | Meso-community | 5280 | 8 |
| MesoMay03 | Mayotte | 12,952667 | 44,970417 | 80 | Low | 2 | Coral | 11800 | 8 |
| MesoMay04 | Mayotte | 12,931583 | 44,96425 | 82 | High | 3 | Meso-community | 12000 | 8 |
| MesoMay05 | Mayotte | 12,817767 | 45,292967 | 89 | Low | 3 | Coral | 600 | 8 |
| MesoMay06 | Mayotte | 12,876 | 45,282517 | 83 | Low | 3 | Coral | 6600 | 8 |
| MesoMay07 | Mayotte | 12,876 | 45,282517 | 83 | Low | 3 | Coral | 6600 | 8 |
| MesoMay08 | Mayotte | 12,974649 | 44,978671 | 76 | High | 3 | Meso-community | 11000 | 8 |
| MesoMay09 | Mayotte | 12,974649 | 44,978671 | 82 | High | 3 | Meso-community | 11000 | 8 |
| MesoMay10 | Mayotte | 12,931583 | 44,96425 | 78 | High | 3 | Meso-community | 12000 | 8 |
