# Supplementary material for: Environmental DNA illuminates the darkness of mesophotic assemblages of fishes from West Indian Ocean
Source: PLoS One. 2025 May 22;20(5):e0322870. doi: 10.1371/journal.pone.0322870 (PMC12097626; doi:10.1371/journal.pone.0322870)
Supplement: S3 Text — The file listed the bioinformatics flowchart followed to assess similarity distribution according to different taxonomic level of divergence. (DOCX) [file pone.0322870.s003.docx]

**Optimum threshold of intra-species divergence for the MiFish 12S amplicon.**

This document details the procedure used to define a genetic distance threshold for performing clustering analyses of article sequences obtained by eDNA, based on the assessment of the barcoding gap calculated on annotated DNA sequences from Genbank. Anlaysis were performed in R software, unless specified otherwise.

**#1. DNA sequences retrievment from Genbank**

Retrieve DNA from genbank using *entrez_search function ()* based on a list of keywoards including « 12S » and a list of 485 fish genus already observed in Reunion and Mayotte.

We retrieved a total of 3672 sequences

**#2. Removing sequence without the amplicon targeted by the Mifish primer set**

Regions corresponding of the two Mifish primers were search (ie 5′- GTCGGTAAAACTCGTGCCAGC -3′ and the complement of 3′- GTTTGACCCTAATCTATGGGGTGATAC -5′) using the vcountPattern function (Biostrings R package, H. Pagès 2017) using five max.mismatch option.

=> A total of 2842 sequences were removed and the customized sequence dataset was composed of 830 DNA sequences. These sequences list was avalaible in the S5 Appendix.

**#3. Remaining sequences were aligned**

After removing the flanking regions of the two primer, multiple sequences alignment were performed online using the *ngphylogeny.fr* website (Dereeper et al. 2008; Lemoine et al. 2019) using the default alignment process available. The output Block Mapping and Gathering with Entropy (BGME) cleaned sequences fasta alignment file (default parameters) were upload and serve for the downstream analysis.

**#4. Pairwise distance were computed between category of sequences**

A total of 344,035 pairwise comparisons were computed using the *dist.dna* function (ape R package, Paradis et Schliep 2019) using the “raw “ model (number of differences divided by the pair base number compared) and pairwise deletion option. Based on sequences name, we categorized the taxonomic level of comparison into six types:

1. Intra species : *Same_Species / Same_Genus / Same_Family / Same_Order / Same_Class*
2. Intra genus : *Different_Species / Same_Genus / Same_Family / Same_Order / Same_Class*
3. Intra family : *Different_Species / Different_Genus / Same_Family / Same_Order / Same_Class*
4. Intra order : *Different_Species / Different_Genus / Different_Family / Same_Order / Same_Class*
5. Intra class : *Different_Species / Different_Genus / Different_Family / Different_Order / Same_Class*
6. Between class : *Different_Species / Different_Genus / Different_Family / Different_Order / Different_Class*

**#5. Optimum threshold analysis**

We used the *threshOpt* function of the SPIDER package (Brown et al. 2012). This returns the total cumulative errors (false positive and false negative) of identification accuracy for different threshold values.

**#6. Results**

The mean genetic distance value observed between intra-species comparisons was 0.0017 (sd 0.0060) and maximum value of 0.1610. The mean genetic distance value observed between inter-species (of the same genus) comparisons was 0.0347 (sd 0.0395) and maximum value of 0.2602. As the taxonomical distance increases, the genetic distance increases, and consequently, the proportion of low taxonomical distances comparisons decreases in benefit of higher taxonomical distance comparisons (Figure 1).


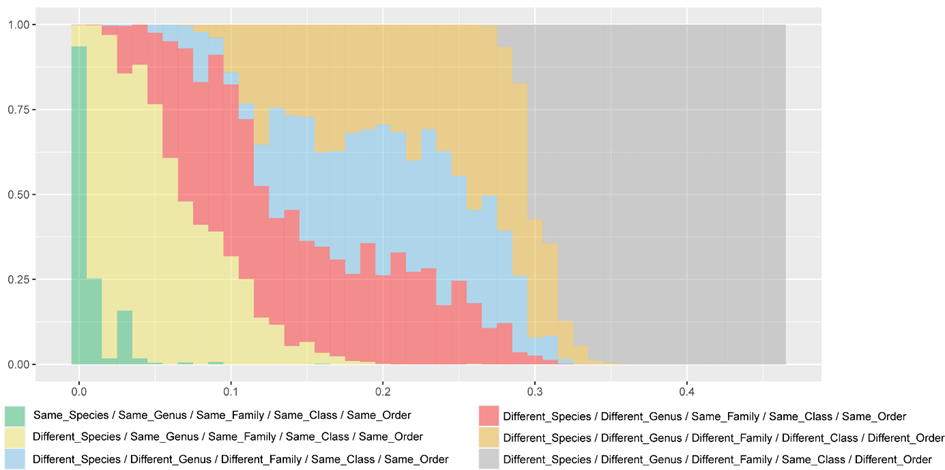


**S3 Fig 1. Relative proportion of taxonomic comparison by genetic distance.** Each type of comparison was characterized by different colors. X-values corresponded to the pairwise genetic distances (with an incrementation of 0.01) between sequences of the customized sequence dataset. The six types of taxonomic comparisons were color-coded. Y-axis corresponded to the proportion of each types of comparison that belonged to the range of the distances value.

Optimum threshold analysis indicated a high range values of ambiguous species identification [378 – 799] depending of the threshold value (Figure 2), with higher rates associated with increased threshold values. A breaking point was observed beyond a threshold value of 0.006, for which ~54% of sequences were correctly identified.

**S3 Fig 2. Optimum thresholds analyses.** Barplot shows the cumulative errors rate of identification of species within the sequence database at thresholds ranging from 0.001 to 0.09. The false-positive are represented by light gray barplots and false-negative by dark gray barplots. The red vertical line characterized the threshold of 0.006 (ie 0.6%).

**References**

Brown, Samuel D. J., Rupert A. Collins, Stephane Boyer, Marie‐Caroline Lefort, Jagoba Malumbres‐Olarte, Cor J. Vink, et Robert H. Cruickshank. 2012. « S pider : An R Package for the Analysis of Species Identity and Evolution, with Particular Reference to DNA Barcoding ». *Molecular Ecology Resources* 12 (3): 562‑65. https://doi.org/10.1111/j.1755-0998.2011.03108.x.

Dereeper, A., V. Guignon, G. Blanc, S. Audic, S. Buffet, F. Chevenet, J.-F. Dufayard, et al. 2008. « Phylogeny.fr: robust phylogenetic analysis for the non-specialist ». *Nucleic Acids Research* 36 (suppl_2): W465‑69. https://doi.org/10.1093/nar/gkn180.

Pagès H., P. Aboyoun. 2017. « Biostrings ». Bioconductor. https://doi.org/10.18129/B9.BIOC.BIOSTRINGS.

Lemoine, Frédéric, Damien Correia, Vincent Lefort, Olivia Doppelt-Azeroual, Fabien Mareuil, Sarah Cohen-Boulakia, et Olivier Gascuel. 2019. « NGPhylogeny.Fr: New Generation Phylogenetic Services for Non-Specialists ». *Nucleic Acids Research* 47 (W1): W260‑65. https://doi.org/10.1093/nar/gkz303.

Paradis, Emmanuel, et Klaus Schliep. 2019. « Ape 5.0: An Environment for Modern Phylogenetics and Evolutionary Analyses in R ». Édité par Russell Schwartz. *Bioinformatics* 35 (3): 526‑28. https://doi.org/10.1093/bioinformatics/bty633.

**R script**

**#Identification of 12S sequences covering the Mifish amplicon from a fasta file “12S sequence fish database”**

my_sequences<-readDNAStringSet("12S sequence fish database.txt")

MiFish_F <- DNAString("GTCGGTAAAACTCGTGCCAGC")

MiFish_R<-DNAString("CAAACTGGGATTAGATACCCCACTATG") #(complement of GTTTGACCCTAATCTATGGGGTGATAC)

Primer_Presence<-data.frame(cbind(

FPresence=vcountPattern(MiFish_F, my_sequences,max.mismatch=5, min.mismatch=0,with.indels=FALSE),

RPresence=vcountPattern(MiFish_R, my_sequences,max.mismatch=5, min.mismatch=0,with.indels=FALSE)))

Primer_Presence$FPresence[Primer_Presence$FPresence==1]<-"Fpresent"

Primer_Presence$RPresence[Primer_Presence$RPresence==1]<-"Rpresent"

Primer_Presence$rule<-paste(Primer_Presence$FPresence,Primer_Presence$RPresence,sep=" and ")

Primer_Presence$Select_number<-c(1:nrow(Primer_Presence))

Select<-subset(Primer_Presence,Primer_Presence$rule=="Fpresent and Rpresent")

DNAset2<-my_sequences[c(Select$Select_number)]

writeXStringSet(DNAset2, file="Amplicon_12S with MiFish v05012025.fasta", format="fasta")

#=> Make sequence alignment (in this study with *ngphylogeny.fr) and removed the flanking region of the MiFish amplicon*

**#Assessing barcoding gap from a alignment file of MiFish amplicon sequence “BMGE_Cleaned_sequences_Fasta”**

fs<-read.FASTA(as.character("BMGE_Cleaned_sequences_Fasta.fasta"), type = "DNA")

doloDist2 <-dist.dna(fs, "raw", pairwise.deletion = TRUE) #"raw model" = is simply the proportion or the number of sites that differ between each pair of sequences.

doloSpp2<-data.frame(str_split(names(fs),"-", simplify=TRUE))

doloSpp3<-paste(doloSpp2$X1,doloSpp2$X2,sep="-")

threshVal <- seq(0.001,0.09, by = 0.001)

opt2 <- lapply(threshVal, function(x) threshOpt(doloDist2, doloSpp3, thresh = x))

optMat2 <- do.call(rbind, opt2)

**#Figure**

barplot(t(optMat2)[4:5,], names.arg=optMat2[,1], xlab="Threshold values",

ylab="Cumulative error")

abline(v = 7.5, col = "red")

legend(x = 1.5, y = 950, legend = c("False positives", "False negatives"),

fill = c("grey75", "grey25"))

**#Clustering ZOTU from a ZOTU sequences alignment fasta file named “MiFish Amplicon alignment v03012025”**

ASV2<-read_fasta("ASV Alignement.FAS", type = "DNA") ##library(metacoder)

ClusEmpiri5<-data.frame(Cluster=seq_cluster(ASV2, threshold = 0.006, method = "complete"))
