# Supplementary material for: Environmental DNA illuminates the darkness of mesophotic assemblages of fishes from West Indian Ocean
Source: PLoS One. 2025 May 22;20(5):e0322870. doi: 10.1371/journal.pone.0322870 (PMC12097626; doi:10.1371/journal.pone.0322870)
Supplement: S4 Table — (DOCX) [file pone.0322870.s004.docx]

**S4 Table 1.** **Results of the homogeneity of dispersion (permutation test, permutest) between islands in the sensitivity analysis.** All tests were computed with one degree of freedom and with 999 permutations.

| **Model** | **Jaccard** | | **Bray-Curtis** | |
| --- | --- | --- | --- | --- |
| Permutest | F | *p* | F | *p* |
| Full | 0.25 | 0.63 | 0.86 | 0.40 |
| Without body shape | 0.05 | 0.82 | 0.51 | 0.50 |
| Without diet | 0.52 | 0.46 | 1.27 | 0.30 |
| Without position | 0.21 | 0.68 | 1.05 | 0.32 |
| Without schooling | 0.39 | 0.54 | 0.45 | 0.59 |
| Without activity | 0.28 | 0.61 | 1.04 | 0.33 |

**S4 Table 2.** **Results of PERMANOVAs between islands in the sensitivity analysis.** All tests were computed with one degree of freedom and with 999 permutations.

| **Model** | **Jaccard** | | | **Bray-Curtis** | | |
| --- | --- | --- | --- | --- | --- | --- |
| PERMANOVA | R² | F | *p* | *R²* | F | *p* |
| Full | 0.15 | 2.85 | 0.001 | 0.22 | 4.52 | 0.001 |
| Without body shape | 0.17 | 3.20 | 0.002 | 0.23 | 4.67 | 0.001 |
| Without diet | 0.14 | 2.56 | 0.003 | 0.22 | 4.46 | 0.001 |
| Without position | 0.16 | 3.06 | 0.002 | 0.23 | 4.66 | 0.002 |
| Without schooling | 0.14 | 2.53 | 0.001 | 0.20 | 4.09 | 0.002 |
| Without activity | 0.14 | 2.62 | 0.002 | 0.21 | 4.21 | 0.002 |
