# Supplementary material for: Environmental DNA illuminates the darkness of mesophotic assemblages of fishes from West Indian Ocean
Source: PLoS One. 2025 May 22;20(5):e0322870. doi: 10.1371/journal.pone.0322870 (PMC12097626; doi:10.1371/journal.pone.0322870)
Supplement: S6 Fig — Four separated analyses were conducted: Fig 1 for Holocentridae; Fig 2 for Muraenidae and Caranguidae; Fig 3 Serranidae and Fig 4 Acanthuridae, Apogonidae and Lutjanidae. The evolutionary history was inferred using the Neighbor-Joining method. The percentage of replicate trees in which the associated taxa clustered together in the bootstrap test (5000 replicates) are shown next to the branches. The evolutionary distances were computed using the number of differences method and are in the units of the number of base differences per sequence. All positions containing gaps and missing data were eliminated (complete deletion option). (PDF) [file pone.0322870.s006.pdf]

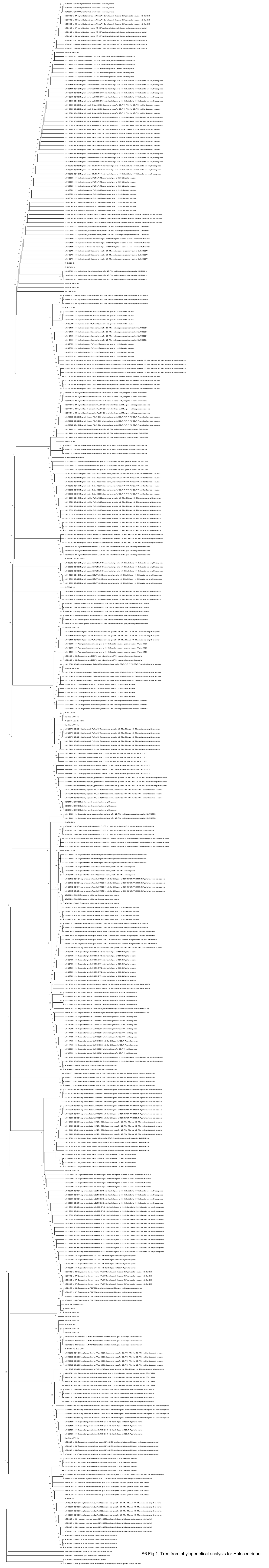

S6 Fig 1. Tree from phylogenetical analysis for Holocentridae.

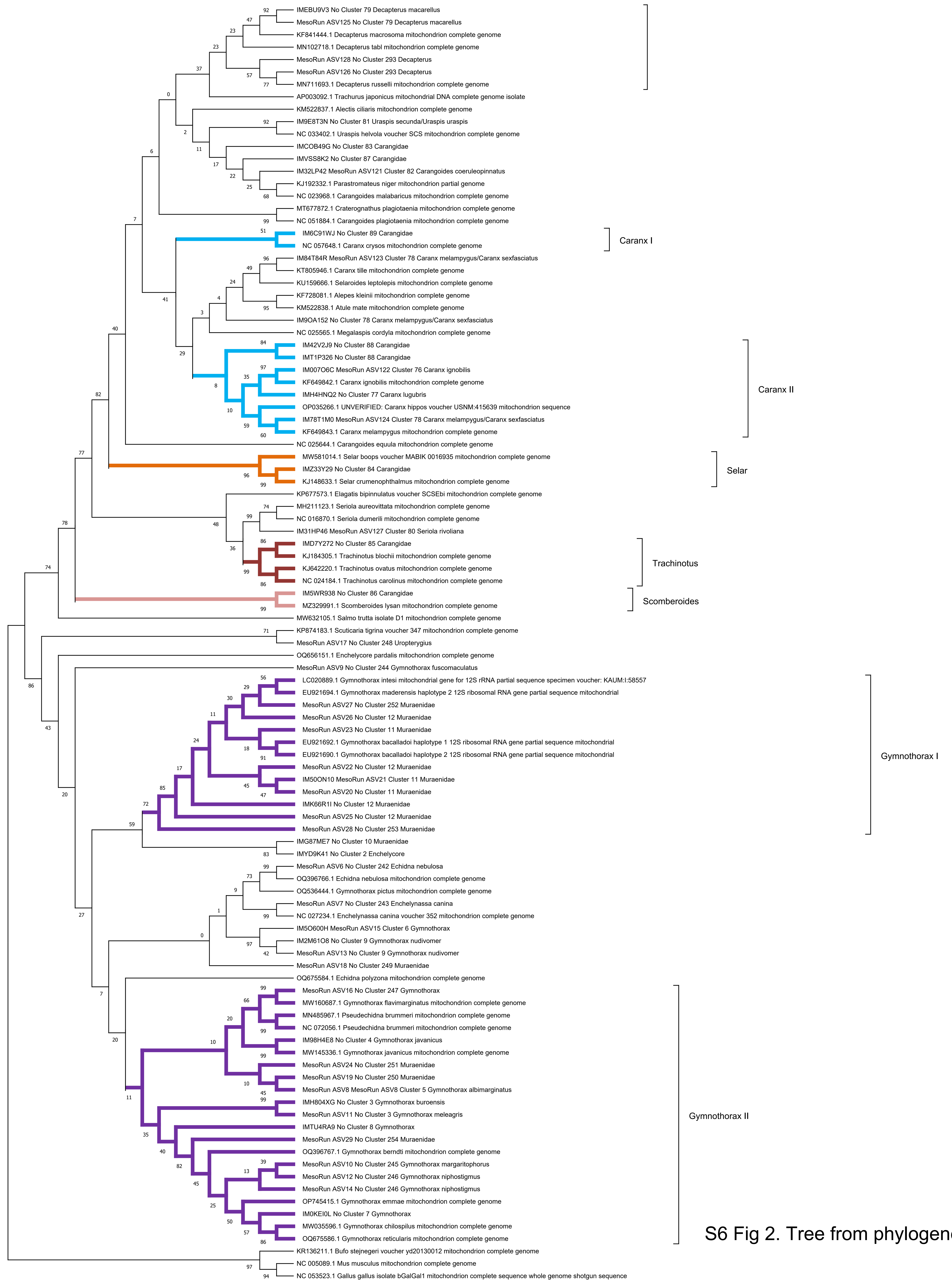

S6 Fig 2. Tree from phylogenetical analysis for Caranguidae and Muraenidae.

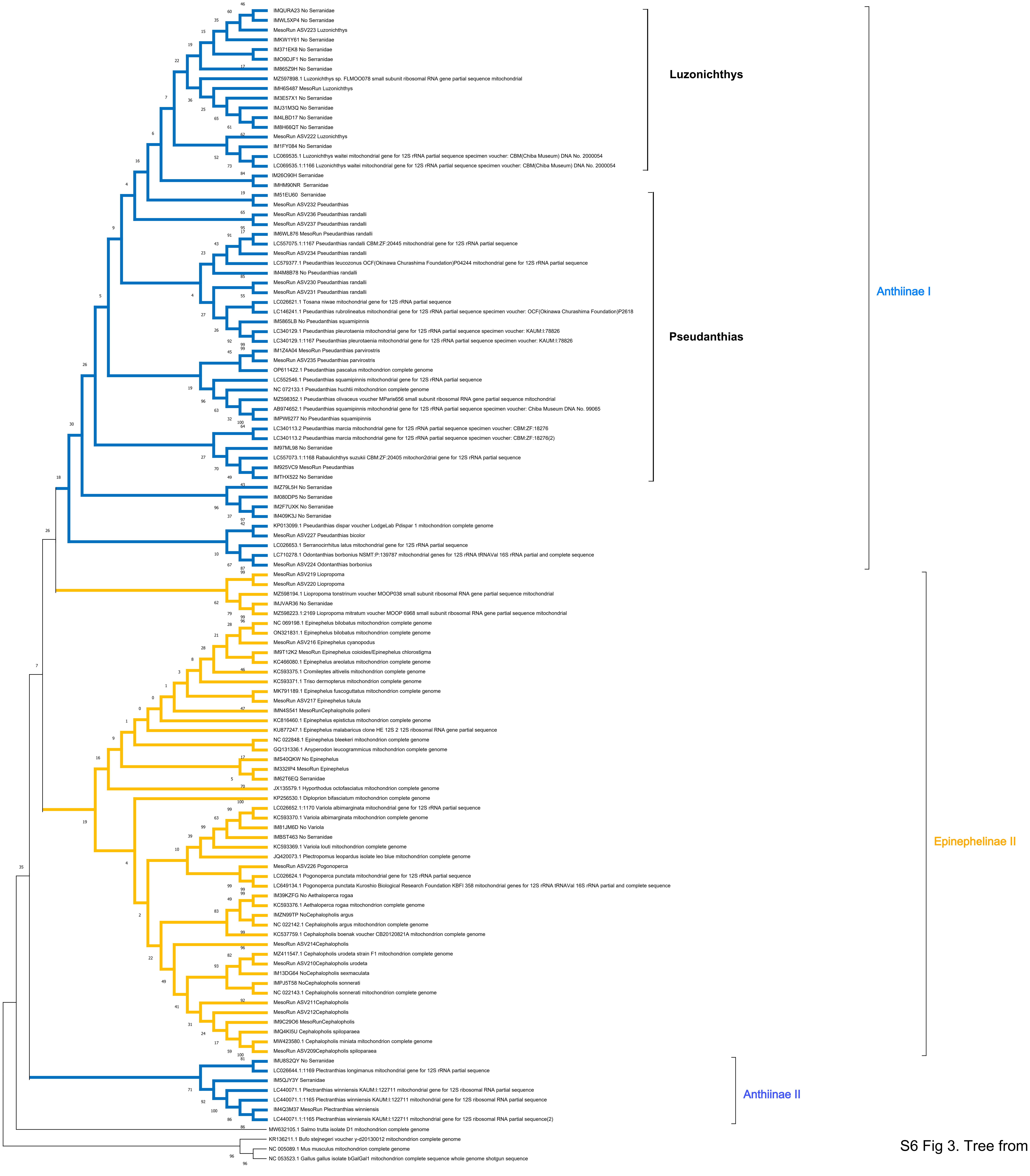

S6 Fig 3. Tree from phylogenetical analysis for Serranidae.

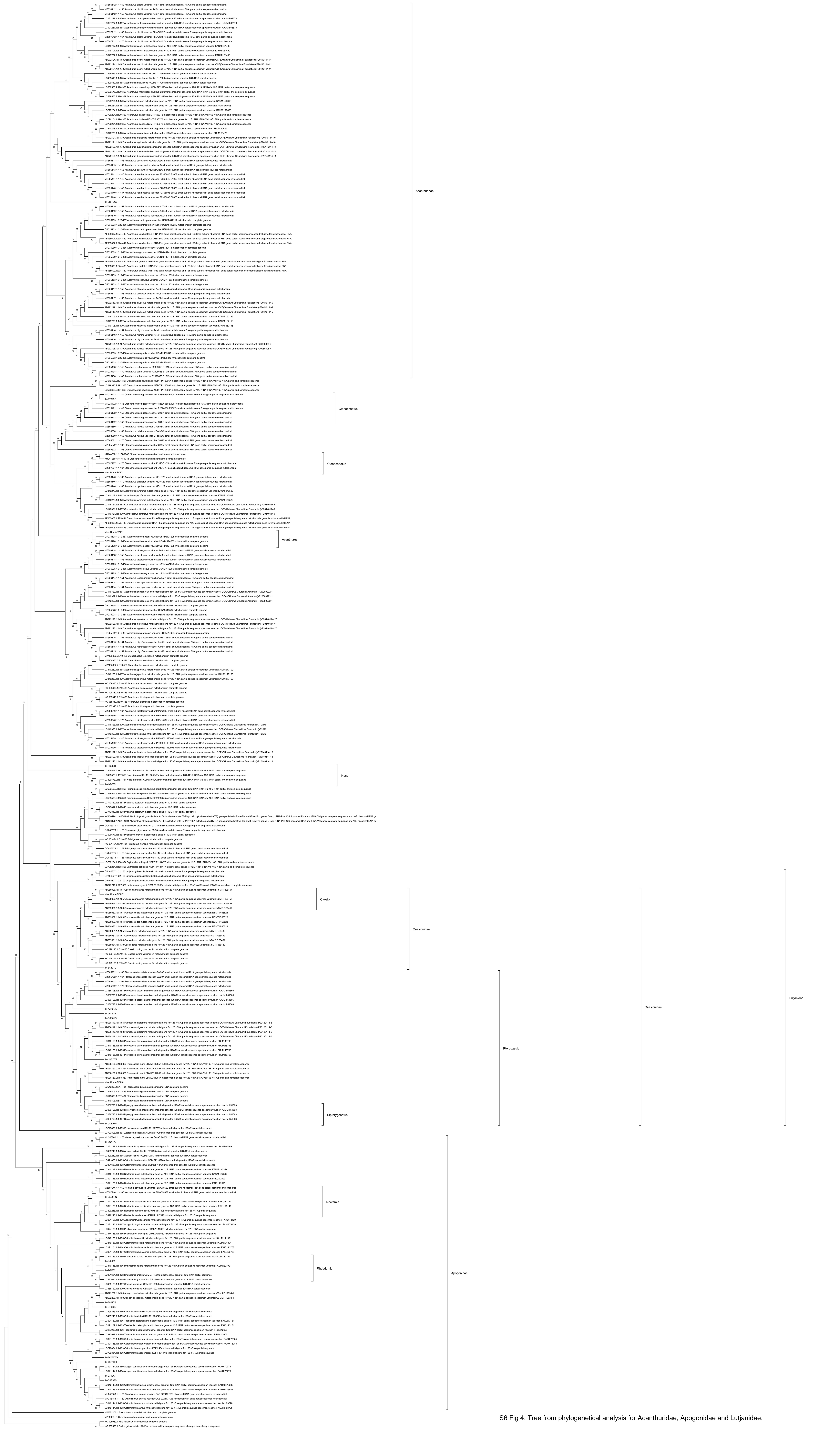

S6 Fig 4. Tree from phylogenetical analysis for Acanthuridae, Apogonidae and Lutjanidae.
