# Supplementary material for: Environmental DNA illuminates the darkness of mesophotic assemblages of fishes from West Indian Ocean
Source: PLoS One. 2025 May 22;20(5):e0322870. doi: 10.1371/journal.pone.0322870 (PMC12097626; doi:10.1371/journal.pone.0322870)
Supplement: S7 Table — This table provides an overview of the conclusive taxonomic assignment ranks of ZOTUs. The “Global” column does not represent cumulative values since certain ZOTUs are shared between the islands. (DOCX) [file pone.0322870.s007.docx]

**S7 Table.** **Summary of the final taxonomic assignment rank of ZOTUs.**

| **Assignement** | **Mayotte** | **La Réunion** | **Global** |
| --- | --- | --- | --- |
| **Species** | **87 (28%)** | **113 (40%)** | **168 (33%)** |
| **Genus** | **129 (43%)** | **128 (46%)** | **229 (44%)** |
| **(Sub-) Family** | **69 (22%)** | **29 (10%)** | **91 (17%)** |
| **Order or above** | **16 (5%)** | **9 (3%)** | **24 (7.6%)** |
|  |  |  |  |
| **Total** | **301** | **279** | **512 (68 shared)** |
