# Supplementary material for: Environmental DNA illuminates the darkness of mesophotic assemblages of fishes from West Indian Ocean
Source: PLoS One. 2025 May 22;20(5):e0322870. doi: 10.1371/journal.pone.0322870 (PMC12097626; doi:10.1371/journal.pone.0322870)
Supplement: S8 Table — This file corresponded to the output of multipatt R function using respectively the taxa grouping (at family level) and functional grouping. (DOCX) [file pone.0322870.s008.docx]

**S8 Table 1.** **Output of *multipatt* R function for families.** Significantly informative families are in bold.

| **Taxa** | **Specificity componant (A)** | | **Sensitivity component (B)** | | **Test** | |
| --- | --- | --- | --- | --- | --- | --- |
| ***Family*** | ***Mayotte*** | ***La Réunion*** | ***Mayotte*** | ***La Réunion*** | ***stat*** | ***p*** |
| Acanthuridae | 0.34 | 0.66 | 0.70 | 1.00 | 0.81 | 0.12 |
| Antennariidae | 0.44 | 0.56 | 0.10 | 0.13 | 0.26 | 1.00 |
| Apogonidae | 0.55 | 0.45 | 1.00 | 0.88 | 0.74 | 0.48 |
| Atherinidae | 0.44 | 0.56 | 0.10 | 0.13 | 0.26 | 1.00 |
| Balistidae | 0.55 | 0.45 | 1.00 | 1.00 | 0.74 | 0.51 |
| Belonidae | 0.44 | 0.56 | 0.10 | 0.13 | 0.26 | 1.00 |
| Blenniidae | 0.21 | 0.79 | 0.10 | 0.38 | 0.54 | 0.28 |
| Bothidae | 0.29 | 0.71 | 0.10 | 0.25 | 0.42 | 0.57 |
| Bramidae | 0.00 | 1.00 | 0.00 | 0.13 | 0.35 | 0.45 |
| Caesionidae | 0.62 | 0.38 | 0.90 | 0.75 | 0.75 | 0.29 |
| Callionymidae | 0.00 | 1.00 | 0.00 | 0.25 | 0.50 | 0.18 |
| **Caranguidae** | **0.66** | **0.34** | **1.00** | **1.00** | **0.81** | **0.03** |
| Chaetodontidae | 0.31 | 0.69 | 0.30 | 0.75 | 0.72 | 0.12 |
| Chlopsidae | 0.00 | 1.00 | 0.00 | 0.25 | 0.50 | 0.18 |
| Cirrhitidae | 0.44 | 0.56 | 0.30 | 0.25 | 0.37 | 1.00 |
| Congridae | 0.17 | 0.83 | 0.10 | 0.38 | 0.56 | 0.22 |
| Coryphaenidae | 0.00 | 1.00 | 0.00 | 0.13 | 0.35 | 0.45 |
| Diodontidae | 0.00 | 1.00 | 0.00 | 0.25 | 0.50 | 0.19 |
| Emmelichthyidae | 0.00 | 1.00 | 0.00 | 0.25 | 0.50 | 0.18 |
| Exocoetidae | 0.00 | 1.00 | 0.00 | 0.38 | 0.61 | 0.07 |
| Fistulariidae | 0.55 | 0.45 | 0.30 | 0.13 | 0.40 | 0.59 |
| Gempylidae | 0.29 | 0.71 | 0.10 | 0.25 | 0.42 | 0.55 |
| Gobiidae | 0.62 | 0.38 | 0.90 | 0.63 | 0.74 | 0.29 |
| Haemulidae | 1.00 | 0.00 | 0.40 | 0.00 | 0.63 | 0.09 |
| Hemiramphidae | 0.00 | 1.00 | 0.00 | 0.25 | 0.50 | 0.18 |
| Holocentridae | 0.40 | 0.60 | 0.80 | 1.00 | 0.78 | 0.15 |
| **Kyphosidae** | **0.12** | **0.88** | **0.10** | **0.63** | **0.74** | **0.04** |
| **Labridae** | **0.66** | **0.34** | **1.00** | **0.88** | **0.81** | **0.04** |
| Lethrinidae | 0.60 | 0.40 | 0.60 | 0.75 | 0.60 | 0.82 |
| Lutjanidae | 0.52 | 0.48 | 1.00 | 1.00 | 0.72 | 0.84 |
| **Malacanthidae** | **0.88** | **0.12** | **0.70** | **0.13** | **0.78** | **0.04** |
| Microdesmidae | 0.78 | 0.22 | 0.80 | 0.38 | 0.79 | 0.05 |
| Monacanthidae | 0.21 | 0.79 | 0.20 | 0.50 | 0.63 | 0.19 |
| Moringuidae | 0.00 | 1.00 | 0.00 | 0.13 | 0.35 | 0.44 |
| Mullidae | 0.37 | 0.63 | 0.60 | 1.00 | 0.80 | 0.08 |
| **Muraenidae** | **0.25** | **0.75** | **0.80** | **1.00** | **0.86** | **0.01** |
| Myctophidae | 0.45 | 0.55 | 0.90 | 0.88 | 0.69 | 0.75 |
| Ophichthidae | 0.35 | 0.65 | 0.20 | 0.25 | 0.40 | 0.80 |
| Ophidiidae | 0.44 | 0.56 | 0.40 | 0.50 | 0.53 | 1.00 |
| Ostraciidae | 0.00 | 1.00 | 0.00 | 0.25 | 0.50 | 0.18 |
| Paralepididae | 1.00 | 0.00 | 0.10 | 0.00 | 0.32 | 1.00 |
| Pempheridae | 1.00 | 0.00 | 0.10 | 0.00 | 0.32 | 1.00 |
| Phosichthyidae | 0.62 | 0.38 | 0.60 | 0.38 | 0.61 | 0.64 |
| Plesiopidae | 1.00 | 0.00 | 0.10 | 0.00 | 0.32 | 1.00 |
| Plotosidae | 1.00 | 0.00 | 0.10 | 0.00 | 0.32 | 1.00 |
| Pomacanthidae | 0.38 | 0.63 | 0.70 | 0.63 | 0.63 | 0.72 |
| Pomacentridae | 0.38 | 0.62 | 0.60 | 0.63 | 0.62 | 0.62 |
| **Priacanthidae** | **0.00** | **1.00** | **0.00** | **0.50** | **0.71** | **0.02** |
| Scaridae | 0.68 | 0.32 | 0.50 | 0.38 | 0.58 | 0.63 |
| Scombridae | 0.40 | 0.60 | 0.60 | 1.00 | 0.77 | 0.10 |
| Scorpaenidae | 0.49 | 0.51 | 0.60 | 0.50 | 0.54 | 1.00 |
| **Serranidae** | **0.63** | **0.37** | **1.00** | **1.00** | **0.79** | **0.00** |
| Sphyraenidae | 0.80 | 0.20 | 0.50 | 0.13 | 0.63 | 0.15 |
| Stomiidae | 0.00 | 1.00 | 0.00 | 0.13 | 0.35 | 0.44 |
| Syngnathidae | 1.00 | 0.00 | 0.10 | 0.00 | 0.32 | 1.00 |
| Synodontidae | 0.37 | 0.63 | 0.70 | 0.88 | 0.74 | 0.30 |
| Tetraodontidae | 0.50 | 0.50 | 0.30 | 0.38 | 0.43 | 0.98 |
| Tripterygiidae | 0.00 | 1.00 | 0.00 | 0.13 | 0.35 | 0.45 |
| Uranoscopidae | 0.00 | 1.00 | 0.00 | 0.13 | 0.35 | 0.45 |
| Xiphiidae | 0.00 | 1.00 | 0.00 | 0.25 | 0.50 | 0.18 |
| **Zanclidae** | **1.00** | **0.00** | **0.50** | **0.00** | **0.71** | **0.03** |

*Note: Specificity refers to the probability that the sampled site belongs to the target island given that the family has been observed. Sensitivity is the probability of detecting the family in samples from a given island.*

**S8 Table 2.** **Output of *multipatt* R function for functional entities (FEs).** Significantly informative FEs are in bold.

| **FE ID** | **Specificity componant (A)** | | **Sensitivity component (B)** | | **Test** | |
| --- | --- | --- | --- | --- | --- | --- |
|  | ***Mayotte*** | ***La Réunion*** | ***Mayotte*** | ***La Réunion*** | ***stat*** | ***p*** |
| **FE_1** | **0.26** | **0.74** | **0.80** | **1.00** | **0.86** | **0.00** |
| **FE_10** | **0.74** | **0.26** | **1.00** | **0.88** | **0.86** | **0.01** |
| FE_100 | 0.00 | 1.00 | 0.00 | 0.25 | 0.50 | 0.19 |
| FE_101 | 0.00 | 1.00 | 0.00 | 0.25 | 0.50 | 0.18 |
| FE_102 | 0.00 | 1.00 | 0.00 | 0.13 | 0.35 | 0.45 |
| FE_103 | 0.00 | 1.00 | 0.00 | 0.13 | 0.35 | 0.45 |
| FE_104 | 0.00 | 1.00 | 0.00 | 0.13 | 0.35 | 0.45 |
| FE_11 | 0.71 | 0.29 | 0.50 | 0.38 | 0.59 | 0.46 |
| FE_12 | 0.39 | 0.61 | 0.80 | 1.00 | 0.78 | 0.14 |
| FE_13 | 0.47 | 0.53 | 0.50 | 0.63 | 0.58 | 0.68 |
| FE_14 | 0.67 | 0.33 | 0.60 | 0.25 | 0.63 | 0.26 |
| FE_15 | 0.17 | 0.83 | 0.20 | 0.63 | 0.72 | 0.11 |
| FE_16 | 0.38 | 0.62 | 0.40 | 0.63 | 0.62 | 0.38 |
| FE_17 | 0.55 | 0.45 | 0.30 | 0.38 | 0.41 | 0.98 |
| FE_18 | 0.83 | 0.17 | 0.40 | 0.13 | 0.58 | 0.26 |
| FE_19 | 0.57 | 0.43 | 0.20 | 0.25 | 0.34 | 1.00 |
| **FE_2** | **0.58** | **0.42** | **1.00** | **1.00** | **0.76** | **0.03** |
| **FE_20** | **0.09** | **0.91** | **0.10** | **0.75** | **0.83** | **0.01** |
| FE_21 | 0.29 | 0.71 | 0.20 | 0.63 | 0.67 | 0.13 |
| FE_22 | 0.41 | 0.59 | 0.40 | 0.75 | 0.67 | 0.31 |
| FE_23 | 0.44 | 0.56 | 0.20 | 0.38 | 0.46 | 0.62 |
| **FE_24** | **0.19** | **0.81** | **0.30** | **0.88** | **0.84** | **0.01** |
| FE_25 | 0.71 | 0.29 | 0.70 | 0.50 | 0.70 | 0.21 |
| FE_26 | 0.74 | 0.26 | 0.50 | 0.25 | 0.61 | 0.30 |
| FE_27 | 0.32 | 0.68 | 0.20 | 0.50 | 0.58 | 0.31 |
| FE_28 | 0.67 | 0.33 | 0.20 | 0.25 | 0.37 | 0.86 |
| FE_29 | 0.57 | 0.43 | 0.40 | 0.38 | 0.48 | 0.84 |
| FE_3 | 0.60 | 0.40 | 0.90 | 0.75 | 0.73 | 0.32 |
| FE_30 | 0.71 | 0.29 | 0.30 | 0.13 | 0.46 | 0.59 |
| FE_31 | 0.29 | 0.71 | 0.30 | 0.50 | 0.60 | 0.33 |
| FE_32 | 0.33 | 0.67 | 0.30 | 0.50 | 0.58 | 0.37 |
| FE_33 | 0.62 | 0.38 | 0.20 | 0.13 | 0.35 | 1.00 |
| **FE_34** | **0.14** | **0.86** | **0.20** | **0.63** | **0.73** | **0.03** |
| FE_35 | 0.80 | 0.20 | 0.50 | 0.13 | 0.63 | 0.15 |
| FE_36 | 0.58 | 0.42 | 0.30 | 0.50 | 0.46 | 0.77 |
| FE_37 | 0.83 | 0.17 | 0.60 | 0.13 | 0.70 | 0.07 |
| **FE_38** | **0.88** | **0.12** | **0.70** | **0.13** | **0.78** | **0.04** |
| **FE_39** | **1.00** | **0.00** | **0.50** | **0.00** | **0.71** | **0.04** |
| FE_4 | 0.46 | 0.54 | 1.00 | 1.00 | 0.73 | 0.49 |
| FE_40 | 0.44 | 0.56 | 0.40 | 0.50 | 0.53 | 1.00 |
| FE_41 | 0.00 | 1.00 | 0.00 | 0.25 | 0.50 | 0.18 |
| **FE_42** | **0.00** | **1.00** | **0.00** | **0.50** | **0.71** | **0.02** |
| **FE_43** | **0.00** | **1.00** | **0.00** | **0.50** | **0.71** | **0.02** |
| FE_44 | 0.62 | 0.38 | 0.20 | 0.13 | 0.35 | 1.00 |
| FE_45 | 0.35 | 0.65 | 0.20 | 0.38 | 0.49 | 0.61 |
| FE_46 | 0.44 | 0.56 | 0.10 | 0.13 | 0.26 | 1.00 |
| FE_47 | 1.00 | 0.00 | 0.70 | 0.00 | 0.84 | 0.00 |
| FE_48 | 1.00 | 0.00 | 0.10 | 0.00 | 0.32 | 1.00 |
| FE_49 | 0.65 | 0.35 | 0.70 | 0.25 | 0.68 | 0.15 |
| FE_5 | 0.45 | 0.55 | 0.80 | 1.00 | 0.74 | 0.35 |
| FE_50 | 0.55 | 0.45 | 0.30 | 0.25 | 0.40 | 1.00 |
| FE_51 | 0.44 | 0.56 | 0.10 | 0.13 | 0.26 | 1.00 |
| **FE_52** | **1.00** | **0.00** | **0.60** | **0.00** | **0.77** | **0.01** |
| FE_53 | 0.80 | 0.20 | 0.50 | 0.13 | 0.63 | 0.16 |
| FE_54 | 0.80 | 0.20 | 0.50 | 0.13 | 0.63 | 0.15 |
| FE_55 | 0.29 | 0.71 | 0.10 | 0.25 | 0.42 | 0.56 |
| FE_56 | 0.44 | 0.56 | 0.10 | 0.13 | 0.26 | 1.00 |
| FE_57 | 0.00 | 1.00 | 0.00 | 0.13 | 0.35 | 0.45 |
| FE_58 | 0.00 | 1.00 | 0.00 | 0.13 | 0.35 | 0.45 |
| FE_59 | 0.44 | 0.56 | 0.10 | 0.13 | 0.26 | 1.00 |
| FE_6 | 0.62 | 0.38 | 0.90 | 0.75 | 0.74 | 0.32 |
| FE_60 | 0.44 | 0.56 | 0.30 | 0.38 | 0.46 | 1.00 |
| FE_61 | 0.41 | 0.59 | 0.60 | 0.88 | 0.72 | 0.32 |
| FE_62 | 0.52 | 0.48 | 0.40 | 0.38 | 0.45 | 1.00 |
| FE_63 | 0.17 | 0.83 | 0.10 | 0.50 | 0.65 | 0.12 |
| **FE_64** | **0.14** | **0.86** | **0.10** | **0.63** | **0.73** | **0.04** |
| FE_65 | 1.00 | 0.00 | 0.20 | 0.00 | 0.45 | 0.48 |
| FE_66 | 1.00 | 0.00 | 0.10 | 0.00 | 0.32 | 1.00 |
| FE_67 | 1.00 | 0.00 | 0.10 | 0.00 | 0.32 | 1.00 |
| FE_68 | 1.00 | 0.00 | 0.10 | 0.00 | 0.32 | 1.00 |
| FE_69 | 1.00 | 0.00 | 0.10 | 0.00 | 0.32 | 1.00 |
| FE_7 | 0.29 | 0.71 | 0.60 | 0.75 | 0.73 | 0.27 |
| FE_70 | 1.00 | 0.00 | 0.10 | 0.00 | 0.32 | 1.00 |
| FE_71 | 1.00 | 0.00 | 0.20 | 0.00 | 0.45 | 0.46 |
| **FE_72** | **1.00** | **0.00** | **0.80** | **0.00** | **0.89** | **0.00** |
| **FE_73** | **0.14** | **0.86** | **0.10** | **0.63** | **0.73** | **0.04** |
| FE_74 | 0.55 | 0.45 | 0.90 | 0.75 | 0.70 | 0.56 |
| FE_75 | 1.00 | 0.00 | 0.10 | 0.00 | 0.32 | 1.00 |
| FE_76 | 1.00 | 0.00 | 0.10 | 0.00 | 0.32 | 1.00 |
| FE_77 | 1.00 | 0.00 | 0.20 | 0.00 | 0.45 | 0.47 |
| FE_78 | 1.00 | 0.00 | 0.10 | 0.00 | 0.32 | 1.00 |
| FE_79 | 0.44 | 0.56 | 0.10 | 0.13 | 0.26 | 1.00 |
| **FE_8** | **0.72** | **0.28** | **1.00** | **1.00** | **0.85** | **0.00** |
| FE_80 | 0.17 | 0.83 | 0.10 | 0.50 | 0.65 | 0.12 |
| FE_81 | 1.00 | 0.00 | 0.20 | 0.00 | 0.45 | 0.49 |
| FE_82 | 1.00 | 0.00 | 0.10 | 0.00 | 0.32 | 1.00 |
| FE_83 | 0.21 | 0.79 | 0.10 | 0.38 | 0.54 | 0.28 |
| FE_84 | 1.00 | 0.00 | 0.20 | 0.00 | 0.45 | 0.48 |
| FE_85 | 0.00 | 1.00 | 0.00 | 0.38 | 0.61 | 0.07 |
| FE_86 | 0.00 | 1.00 | 0.00 | 0.13 | 0.35 | 0.45 |
| FE_87 | 0.00 | 1.00 | 0.00 | 0.25 | 0.50 | 0.18 |
| FE_88 | 0.00 | 1.00 | 0.00 | 0.13 | 0.35 | 0.45 |
| FE_89 | 0.00 | 1.00 | 0.00 | 0.13 | 0.35 | 0.45 |
| FE_9 | 0.63 | 0.38 | 0.90 | 1.00 | 0.75 | 0.36 |
| FE_90 | 0.00 | 1.00 | 0.00 | 0.38 | 0.61 | 0.08 |
| **FE_91** | **0.00** | **1.00** | **0.00** | **0.50** | **0.71** | **0.02** |
| FE_92 | 0.00 | 1.00 | 0.00 | 0.13 | 0.35 | 0.44 |
| FE_93 | 0.00 | 1.00 | 0.00 | 0.13 | 0.35 | 0.45 |
| FE_94 | 0.00 | 1.00 | 0.00 | 0.13 | 0.35 | 0.44 |
| FE_95 | 0.00 | 1.00 | 0.00 | 0.13 | 0.35 | 0.45 |
| FE_96 | 0.00 | 1.00 | 0.00 | 0.13 | 0.35 | 0.45 |
| FE_97 | 0.00 | 1.00 | 0.00 | 0.25 | 0.50 | 0.18 |
| FE_98 | 0.00 | 1.00 | 0.00 | 0.13 | 0.35 | 0.45 |
| FE_99 | 0.00 | 1.00 | 0.00 | 0.13 | 0.35 | 0.45 |

*Note: Specificity refers to the probability that the sampled site belongs to the target island given that the FE has been observed. Sensitivity is the probability of detecting the FE in samples from a given island.*
