# Supplementary material for: Environmental DNA illuminates the darkness of mesophotic assemblages of fishes from West Indian Ocean
Source: PLoS One. 2025 May 22;20(5):e0322870. doi: 10.1371/journal.pone.0322870 (PMC12097626; doi:10.1371/journal.pone.0322870)
Supplement: S9 Table — (DOCX) [file pone.0322870.s009.docx]

**S9 Table.** **List of combined trait values for each functional entity (FE).** The significant informative FEs based on the *multipatt* tests were indicated by *. Invertivorous mobile inv.: invertivorous fish primarily targeting mobile invertebrates and invertivorous sessile inv.: invertivorous fish primarily targeting sessile invertebrates.

| **FE ID** | **Activity** | **Schooling** | **Position** | **Diet** | **Body shape** | **Significant informative FE** |
| --- | --- | --- | --- | --- | --- | --- |
| FE_1 | nocturnal | solitary | benthic | piscivorous | eel-like | * |
| FE_2 | diurnal | [3 ; 20[ | bentho-pelagic | planktivorous | fusiform/normal | * |
| FE_3 | diurnal | solitary | benthic | invertivorous mobile inv. | fusiform/normal |  |
| FE_4 | both | solitary | benthic | piscivorous | fusiform/normal |  |
| FE_5 | nocturnal | [3 ; 20[ | bentho-pelagic | planktivorous | fusiform/normal |  |
| FE_6 | diurnal | >50 | pelagic | planktivorous | fusiform/normal |  |
| FE_7 | diurnal | [3 ; 20[ | benthic | herbivorous-detritivorous | short and/or deep |  |
| FE_8 | diurnal | [20 ; 50[ | bentho-pelagic | planktivorous | fusiform/normal | * |
| FE_9 | diurnal | solitary | benthic | invertivorous mobile inv. | short and/or deep |  |
| FE_10 | both | [3 ; 20[ | pelagic | piscivorous | fusiform/normal | * |
| FE_11 | nocturnal | [20 ; 50[ | bentho-pelagic | planktivorous | short and/or deep |  |
| FE_12 | diurnal | [20 ; 50[ | bentho-pelagic | planktivorous | short and/or deep |  |
| FE_13 | diurnal | [3 ; 20[ | benthic | invertivorous mobile inv. | fusiform/normal |  |
| FE_14 | diurnal | solitary | benthic | omnivorous | fusiform/normal |  |
| FE_15 | nocturnal | solitary | benthic | invertivorous mobile inv. | eel-like |  |
| FE_16 | nocturnal | solitary | bentho-pelagic | invertivorous mobile inv. | fusiform/normal |  |
| FE_17 | nocturnal | [20 ; 50[ | bentho-pelagic | piscivorous | fusiform/normal |  |
| FE_18 | nocturnal | solitary | benthic | invertivorous mobile inv. | fusiform/normal |  |
| FE_19 | diurnal | [3 ; 20[ | benthic | omnivorous | fusiform/normal |  |
| FE_20 | diurnal | pairing | benthic | invertivorous mobile inv. | short and/or deep | * |
| FE_21 | diurnal | pairing | benthic | invertivorous sessile inv. | short and/or deep |  |
| FE_22 | diurnal | [20 ; 50[ | pelagic | planktivorous | fusiform/normal |  |
| FE_23 | both | solitary | benthic | piscivorous | short and/or deep |  |
| FE_24 | both | solitary | benthic | piscivorous | elongated | * |
| FE_25 | nocturnal | [3 ; 20[ | bentho-pelagic | piscivorous | fusiform/normal |  |
| FE_26 | diurnal | [3 ; 20[ | benthic | omnivorous | short and/or deep |  |
| FE_27 | both | [20 ; 50[ | benthic | invertivorous mobile inv. | fusiform/normal |  |
| FE_28 | diurnal | [20 ; 50[ | pelagic | piscivorous | fusiform/normal |  |
| FE_29 | diurnal | pairing | bentho-pelagic | planktivorous | elongated |  |
| FE_30 | diurnal | solitary | benthic | omnivorous | short and/or deep |  |
| FE_31 | diurnal | [3 ; 20[ | pelagic | piscivorous | elongated |  |
| FE_32 | both | solitary | pelagic | piscivorous | fusiform/normal |  |
| FE_33 | nocturnal | solitary | benthic | piscivorous | fusiform/normal |  |
| FE_34 | nocturnal | solitary | bentho-pelagic | invertivorous mobile inv. | short and/or deep | * |
| FE_35 | both | pairing | benthic | piscivorous | elongated |  |
| FE_36 | diurnal | >50 | pelagic | piscivorous | fusiform/normal |  |
| FE_37 | diurnal | solitary | benthic | invertivorous sessile inv. | short and/or deep |  |
| FE_38 | diurnal | pairing | bentho-pelagic | invertivorous mobile inv. | elongated | * |
| FE_39 | nocturnal | [20 ; 50[ | pelagic | piscivorous | elongated | * |
| FE_40 | nocturnal | solitary | bentho-pelagic | piscivorous | eel-like |  |
| FE_41 | both | solitary | pelagic | piscivorous | elongated |  |
| FE_42 | diurnal | pairing | benthic | herbivorous-detritivorous | short and/or deep | * |
| FE_43 | nocturnal | [3 ; 20[ | bentho-pelagic | invertivorous mobile inv. | short and/or deep | * |
| FE_44 | diurnal | pairing | benthic | invertivorous mobile inv. | fusiform/normal |  |
| FE_45 | diurnal | [20 ; 50[ | benthic | omnivorous | fusiform/normal |  |
| FE_46 | both | [3 ; 20[ | pelagic | piscivorous | elongated |  |
| FE_47 | diurnal | [20 ; 50[ | bentho-pelagic | planktivorous | elongated | * |
| FE_48 | nocturnal | [20 ; 50[ | bentho-pelagic | planktivorous | fusiform/normal |  |
| FE_49 | both | [3 ; 20[ | bentho-pelagic | piscivorous | fusiform/normal |  |
| FE_50 | diurnal | solitary | bentho-pelagic | invertivorous mobile inv. | short and/or deep |  |
| FE_51 | diurnal | [20 ; 50[ | benthic | macroalgal herbivorous | fusiform/normal |  |
| FE_52 | diurnal | solitary | benthic | piscivorous | fusiform/normal | * |
| FE_53 | both | [20 ; 50[ | meso-bathypelagic | piscivorous | fusiform/normal |  |
| FE_54 | diurnal | solitary | benthic | invertivorous mobile inv. | elongated |  |
| FE_55 | diurnal | >50 | benthic | herbivorous-detritivorous | short and/or deep |  |
| FE_56 | nocturnal | [20 ; 50[ | benthic | invertivorous mobile inv. | fusiform/normal |  |
| FE_57 | both | >50 | bentho-pelagic | planktivorous | fusiform/normal |  |
| FE_58 | diurnal | [3 ; 20[ | benthic | invertivorous sessile inv. | short and/or deep |  |
| FE_59 | diurnal | [20 ; 50[ | benthic | invertivorous mobile inv. | fusiform/normal |  |
| FE_60 | nocturnal | >50 | benthic | invertivorous mobile inv. | fusiform/normal |  |
| FE_61 | nocturnal | >50 | bentho-pelagic | invertivorous mobile inv. | fusiform/normal |  |
| FE_62 | nocturnal | solitary | bentho-pelagic | piscivorous | fusiform/normal |  |
| FE_63 | both | [3 ; 20[ | pelagic | piscivorous | short and/or deep |  |
| FE_64 | diurnal | [3 ; 20[ | pelagic | piscivorous | fusiform/normal | * |
| FE_65 | nocturnal | [20 ; 50[ | bentho-pelagic | invertivorous mobile inv. | short and/or deep |  |
| FE_66 | diurnal | pairing | bentho-pelagic | planktivorous | short and/or deep |  |
| FE_67 | diurnal | solitary | benthic | invertivorous mobile inv. | eel-like |  |
| FE_68 | diurnal | solitary | benthic | omnivorous | elongated |  |
| FE_69 | diurnal | [20 ; 50[ | bentho-pelagic | omnivorous | short and/or deep |  |
| FE_70 | both | [20 ; 50[ | bentho-pelagic | invertivorous mobile inv. | fusiform/normal |  |
| FE_71 | both | [20 ; 50[ | bentho-pelagic | invertivorous mobile inv. | short and/or deep |  |
| FE_72 | diurnal | [20 ; 50[ | benthic | planktivorous | fusiform/normal | * |
| FE_73 | diurnal | [20 ; 50[ | bentho-pelagic | macroalgal herbivorous | fusiform/normal | * |
| FE_74 | nocturnal | pairing | bentho-pelagic | invertivorous mobile inv. | elongated |  |
| FE_75 | diurnal | [3 ; 20[ | bentho-pelagic | planktivorous | short and/or deep |  |
| FE_76 | both | [3 ; 20[ | pelagic | invertivorous mobile inv. | fusiform/normal |  |
| FE_77 | nocturnal | [3 ; 20[ | benthic | invertivorous mobile inv. | fusiform/normal |  |
| FE_78 | diurnal | >50 | pelagic | planktivorous | elongated |  |
| FE_79 | diurnal | [20 ; 50[ | pelagic | planktivorous | short and/or deep |  |
| FE_80 | nocturnal | [3 ; 20[ | bentho-pelagic | invertivorous mobile inv. | fusiform/normal |  |
| FE_81 | diurnal | >50 | bentho-pelagic | planktivorous | fusiform/normal |  |
| FE_82 | both | solitary | benthic | piscivorous | eel-like |  |
| FE_83 | diurnal | [20 ; 50[ | benthic | omnivorous | short and/or deep |  |
| FE_84 | diurnal | [3 ; 20[ | benthic | macroalgal herbivorous | short and/or deep |  |
| FE_85 | nocturnal | pairing | bentho-pelagic | planktivorous | short and/or deep |  |
| FE_86 | diurnal | solitary | benthic | herbivorous-detritivorous | elongated |  |
| FE_87 | diurnal | solitary | benthic | herbivorous-detritivorous | fusiform/normal |  |
| FE_88 | both | [3 ; 20[ | meso-bathypelagic | piscivorous | elongated |  |
| FE_89 | diurnal | [20 ; 50[ | bentho-pelagic | omnivorous | fusiform/normal |  |
| FE_90 | nocturnal | [20 ; 50[ | pelagic | planktivorous | fusiform/normal |  |
| FE_91 | diurnal | [3 ; 20[ | pelagic | invertivorous mobile inv. | fusiform/normal | * |
| FE_92 | nocturnal | [20 ; 50[ | bentho-pelagic | invertivorous mobile inv. | fusiform/normal |  |
| FE_93 | both | [3 ; 20[ | pelagic | planktivorous | short and/or deep |  |
| FE_94 | nocturnal | [3 ; 20[ | pelagic | piscivorous | elongated |  |
| FE_95 | diurnal | solitary | benthic | planktivorous | fusiform/normal |  |
| FE_96 | both | solitary | benthic | invertivorous sessile inv. | short and/or deep |  |
| FE_97 | nocturnal | solitary | benthic | herbivorous-detritivorous | short and/or deep |  |
| FE_98 | both | solitary | benthic | invertivorous mobile inv. | short and/or deep |  |
| FE_99 | both | solitary | bentho-pelagic | planktivorous | short and/or deep |  |
| FE_100 | diurnal | [3 ; 20[ | benthic | planktivorous | eel-like |  |
| FE_101 | nocturnal | solitary | meso-bathypelagic | invertivorous mobile inv. | short and/or deep |  |
| FE_102 | nocturnal | solitary | benthic | piscivorous | short and/or deep |  |
| FE_103 | diurnal | solitary | benthic | piscivorous | eel-like |  |
| FE_104 | diurnal | solitary | bentho-pelagic | herbivorous-detritivorous | short and/or deep |  |
